# Domestic Summary

**Published:** 1838-09-12

**Authors:** 


### DOMESTIC SUMMARY.

*Select Medical Library, and Eclectic Journal of Medicine.*—The number for the current month of this excellent medical publication, edited by Dr. John Bell, contains the commencement of *Andral's Clinique*, in an English dress. This is certainly one of the most valuable and practical of the French works, and has enjoyed so great a reputation since its appearance, a few years ago, that it has been a matter of surprise to us that no translation and republication of it has been attempted in this country before. No physician should be without it.

*Dr. John C. Warren.*—It is with great pleasure that we announce the return of Dr. Warren, from his visit to Europe. While abroad, Dr. W. received marked and flattering attention, not only from men eminent in the profession, but also from persons distinguished in scientific and literary pursuits; and we need hardly say, that his return to this country will be warmly welcomed by all who respect (and who does not?) high moral excellence, united with professional attainments not excelled, if equalled, by any.—*Bost. Med. and Surg. Journ.*, Sept. 5.

*Successful Amputation of nearly one-half of the Lower Jaw-Bone, (four and three-quarter inches, including one of its angles,) for Osteosarcoma.* By Paul F. Eve, M. D., Professor of Surgery in the Medical College of Georgia.

My attention was first called to the following case, about the middle of last May, by my friend Dr. Philip S. Lemle, a highly intelligent practitioner of medicine, of Louisville, in this state. The patient is a negro woman, about twenty-five years of age, the mother of one child; she had experienced pain in the left side of the lower jaw-bone for ten years. Some of her friends think that she had suffered even from childhood, what was supposed the tooth-ach. The molar and bicuspid teeth of the side affected had all been successively removed, the last by Dr. Lemle, about four months before the operation. A very large tumour had gradually developed itself around the left half of the lower jaw-bone, and as it was at one time somewhat elastic at one point, had been punctured, from which, however, there flowed only a few drops of blood.

Dinah, the patient, was brought to Augusta on the 26th of last May, and placed under the care of Dr. Antony and myself. In a letter addressed to us, it was stated, "that she had been complaining for years of the jaw-ach, which had entirely resisted the usual remedies for the tooth-ach. The presumption, therefore, is, that the disease has been gradually working its ravages for a great length of time." We were particularly instructed under no circumstances to operate, without there existed a *reasonable hope* of saving her life. It was first determined by us in consultation, to prepare the patient for an operation, which had been decided upon, not only from the existing circumstances of the case, but also from the knowledge of the judicious treatment of the disease by Dr. Lemle, aided by Dr. Jenkins, an old and very respectable physician, also of Louisville. But during the night of the 29th, three days after her arrival, Dinah was nearly suffocated by the pressure of the tumour upon the larynx, and was only able to swallow after the application of ice to it. This at once hastened our preparations for the operation, which was performed on the 31st of May, and certainly not under very favourable circumstances.

Assisted by the faculty, but more especially by Drs. Antony and Newton, the operation was commenced by making an incision from the left angle of the mouth, and extending it in a perpendicular line to the thyroid gland, from which an elliptical one was made to the lobe of the left ear, including the most prominent part of the tumour in the ellipsis. Upon cutting through the lip and denuding the lower jaw-bone, we found an effort of nature at separation near its symphysis. Extracting the canine or stomach tooth, the bone was divided by a small saw, half an inch beyond the line marked by the absorbents. The next object was the removal of the inferior maxillary on the affected side from its connection with the temporal bone, or of its division, provided the disease was arrested in it short of this articulation. By careful dissection, a line was perceived and defined by the absorbents in the lower part of its neck. The saw was again employed, leaving only the condyle with a small portion of the neck, and the operation was completed by detaching the insertion of the temporal muscle into the coronoid process of this bone, which was removed with the diseased mass. The section of the lower jaw-bone measured at its base four and three-quarter inches.

The outer surface of the portion of bone removed, was very rough and denuded of its periosteum, to which latter was attached a large irregular fungous growth, varying in consistency from cartilage to fibrous structure, and extending into the skin and surrounding tissues—there being nothing in this direction like a cyst or even decided limit to the disease. The periosteum of the inner surface of the bone was not completely detached from it, and to it were also adherent large masses of fungus, which had filled the mouth, pushing the tongue to the right side, and projecting down the throat. These had an investing membrane of a delicate structure, and resembled large irregular tubercles. The artery of the lower jaw-bone was entirely obliterated, and its canal was greatly enlarged and made very rough by the action of the absorbents. At both the divisions, however, made by the saw, this bone bled freely, thereby proving that at these places it was sound and unaffected by the disease which had destroyed a portion of its body.

As the patient had fainted several times during the operation, though sustained by stimuli, and as the tumour was not encysted, it was found impracticable to remove every part which had become affected by the diseased action. We had, moreover, proceeded in this case upon the principle, that the disease originated in the bone, and that if the root and body of the tumour were extracted, its projections into the surrounding tissues would necessarily be absorbed. A small tubercle was, therefore, left under the zygomatic arch, together with some enlargement in the skin over the left carotid artery, and also over the thyroid cartilage.

The application of three ligatures to as many arteries, some eight or ten sutures in the skin, with adhesive strips and patent lint to fill up the cavity made by the removal of the jaw-bone and tumour, with a bandage, completed the dressing; and the patient was placed in bed, after having been on the operating table three hours. Much of this time, however, was consumed in restoring her from syncope.

At 8½ o'clock, seven hours after the operation, found the system of the patient re-acting. Took at 4½, a tea-spoonful of common solution of morphine, which afforded much relief, and was swallowed with ease.

*June 1st*, 5 A. M.—Had a pretty good night; drank freely of cold water—nothing else. Prescribed chlorine tooth-wash for mouth. 8 A. M.—Pulse one hundred and twenty-four. Took a table-spoonful of gruel, not relished; sick at stomach; quiet remaining part of this day.

*June 2d*, 3½ A. M.—Cannot swallow; fever. Prescribed ice water, warm pediluvium; head to be elevated. 8 A. M.—Pulse one hundred and twenty-four; deglutition easy. 12 M.—Has slept quietly, and desires nourishment; prescribed gruel. 4 P. M.—Pulse one hundred and forty.

*June 3d*, 2 A. M.—Called to patient on account of sick stomach; prescribed enema of salt and water, morphine and free use of chloride of soda to mouth. 8 A. M.—Pulse one hundred and twenty; patient comfortable. 2 P. M.—Dressed the wound, took off all the plasters; looks well; patient sitting up. 7 P. M.—Pulse one hundred and twenty; took a cup of tea.

*June 4th*, 7 A. M.—Pulse one hundred and eighteen; patient assists herself in bed, and sits up. 5 P. M.—Has appetite; pulse the same. (It has now rained almost incessantly for the last fifty hours.)

*June 5th*, 7 A. M.—Pulse one hundred and four. Dressed the wound, and continued to do so every other day. Removed to-day all the sutures. Union by the first intention took place at the lip and near the lobe of the ear. The skin in the angle of the wound near the thyroid gland sloughed, and at one or two other points where the stitches had been applied. The patient gradually improved; granulations commenced on the ninth day after the operation; and on the tenth, Dinah walked out of her room.

I have nothing particular to relate concerning the patient up to the 17th, except the difficulty, common with all negroes, of making her comprehend the importance of diet. She would insist upon solid food, particles of which were frequently found in the lips of the wound. She had also two attacks of colic, the result of eating improperly. It was about this time I perceived the skin taking on disease in the region of the pomum adami, and soon two tubercles projected from it into the wound, all of which had cicatrized except this place, where an opening was still kept up, and through which a portion of her ingesta, particularly fluids, would flow out.

On the 21st of June, I had to leave Augusta for Charleston, to bring home a near relative, saved from the awful shipwreck of the Pulaski; and on my return, saw with regret, that diseased action, apparently of the most malignant nature, had not only commenced in the skin, but had also invaded the sound cicatrix. Kreosote, iodine, &c., were now freely employed, but seemingly to little purpose, and Dinah left on the 9th of July for the country.

I had the pleasure to hear on the 3d of August, (more than two months since the operation,) from my patient, who is unexpectedly much improved. She has still continued the internal use of iodine, nine drops of the tincture three times daily, and dresses the ulcer with chloride of soda. I learn the diseased skin has sloughed off, and the only tumefaction now existing is in the right submaxillary gland. There is no enlargement under the zygomatic arch, nor in the course of the left carotid. Her appetite is good, and she takes exercise daily.

